# Seventeen-millimeter St. Jude Medical Regent valve in patients with small aortic annulus: dose moderate prosthesis-patient mismatch matter?

**DOI:** 10.1186/1749-8090-9-17

**Published:** 2014-01-17

**Authors:** Jia Hu, Hong Qian, Ya-jiao Li, Jun Gu, Jing Janice Zhao, Er-yong Zhang

**Affiliations:** 1 Department of Cardiovascular Surgery, West China Hospital, Sichuan University, Chengdu, People’s Republic of China; 2 Department of Cardiology, West China Hospital, Sichuan University, Chengdu, People’s Republic of China

**Keywords:** Prosthesis-patient mismatch, 17-mm Regent valve, Aortic valve replacement, Clinical outcome

## Abstract

**Background:**

The study was designed to evaluate the effects of moderate prosthesis-patient mismatch (defined as 0.65 cm^2^/m^2^ < indexed effective orifice area ≤ 0.85 cm^2^/m^2^) on midterm outcomes after isolated aortic valve replacement with a 17-mm St. Jude Medical Regent valve in a large series of patients, and to determine if these effects are influenced by patient confounding variables.

**Methods:**

One-hundred and six patients with and without moderate prosthesis-patient mismatch early after implantation of a 17-mm Regent valve at aortic position were included. Both clinical and echocardiographic assessments were performed preoperatively, at discharge and during follow-up period (mean follow-up time 52.6 ± 11.9 months).

**Results:**

The prevalence of moderate prosthesis-patient mismatch was documented in 46 patients (43.4%) at discharge. During the follow-up period, no difference in the regression of left ventricular mass, decrease of transvalvular pressure gradients, mortality and prosthesis-related complications was observed between patients with and without moderate prosthesis-patient mismatch. After adjustment for several risk factors, moderate prosthesis-patient mismatch was associated with increased midterm mortality in patients with baseline left ventricular ejection fraction < 50% (HR: 1.80, p = 0.02), but with normal prognosis in those with preserved LV function. Younger age (cut off value = 65 years) was not an independent predictor of increased midterm mortality and valve-related complications in patients with moderate prosthesis-patient mismatch.

**Conclusions:**

Moderate prosthesis-patient mismatch after aortic valve replacement with a small mechanical prosthesis is associated with increased mortality and adverse events in patients with pre-existing left ventricular dysfunction. Selected patients with small aortic annulus can experience satisfactory clinical improvements and midterm survival after aortic valve replacement with a 17-mm Regent valve.

## Background

Dealing with a small aortic root of less than 19 mm during aortic valve replacement (AVR) remains a challenging scenario for the cardiac surgeons with regard to operative techniques and selection of appropriate prosthesis [1-4]. As implantation of a smaller prosthetic valve without adequate effective orifice area index (EOAi) may predispose to unfavorable outcomes, several strategies at the time of operation have been developed to avoid the feared prosthesis-patient mismatch (P-PtM, defined as EOAi ≤ 0.85 cm^2^/m^2^) [5-8], including the use of aortic root enlargement/replacement procedures with a larger prosthesis (eg. a stentless bioprosthesis or a new generation of stented bioprosthesis) or modern bileaflet mechanical valve implanted in the supra-annular position. However, these strategies are technically more difficult, require longer cardiopulmonary bypass times, and fail to point uniformly toward an improved clinical outcome [2,3].

The 17-mm St. Jude Medical Regent (SJMR) valve with an improved hemodynamic profile is regarded as a valid option for patients who have small aortic roots [9,10]. Although the Regent valves offer a larger EOA than that of conventional prosthetic valves, the occurrence of P-PtM in patients with 17-mm SJMR valves is reported as high as 31%-94.1% [1,2,9-11]. The main hemodynamic consequence of P-PtM, especially the severe P-PtM (EOAi ≤ 0.65 cm^2^/m^2^), is to generate higher than expected transvalvular gradients, which is responsible for incomplete left ventricular mass (LVM) regression, increased valve-related complications and postoperative mortality [6,7,12-15]. However, controversies still exist as to the impacts of early postoperative moderate P-PtM (0.65 cm^2^/m^2^ < EOAi ≤ 0.85 cm^2^/m^2^) on late outcomes [5-7,12-15]. Moreover, reported experience with 17-mm Regent valves in a large series of patients are rare. Thus, the objective of this study was to evaluate the effects of moderate P-PtM on midterm outcomes after isolated AVR with a 17-mm SJMR valve and to determine if these effects are modulated by patient’s confounding variables.

## Methods

### Patient profile

From December 2006 and June 2012, consecutive 114 patients underwent isolated AVR with a 17-mm SJMR valve in our hospital. Moderate P-PtM (0.65 cm^2^/m^2^ < EOAi ≤ 0.85 cm^2^/m^2^) was present in 106 patients and severe P-PtM (EOAi <0.65 cm^2^/m^2^) in 8 patients. All the data were prospectively collected and retrospectively analyzed. One hundred and six patients were divided into two subsets according to the presence of moderate P-PtM at discharge. The study group comprised 79 women (74.5%) and 27 men (25.5%) with a mean age of 52.5 ± 8.6 years. Mean body surface area (BSA) was 1.56 ± 0.16 m^2^. Fifty-four patients (50.9%) were in New York Heart Functional (NYHA) class III/IV preoperatively. Our investigation, including follow-up studies, was approved by the Ethical Review Board of West China Hospital in compliance with the Declaration of Helsinki. All patients had previously granted permission for the anonymous use of their medical information. The demographic and clinical data of all patients are summarized in Table 1.

**Table 1:** **Baseline clinical characteristics and operative data in patients with and without moderate prosthesis**-**patient mismatch** (**P**-**PtM**)

**Variables**	**Overall**	**Non-PPM**	**Moderate PPM**	*p **value**
	**(n = 106)**	**(n = 58)**	**(n = 45)**	
Age, years	52.5 ± 8.6	52.1 ± 10.2	53.0 ± 12.7	0.398
Age group, years				
≤ 65	56(52.8%)	30(51.7%)	25(55.6%)	0.831
> 65	50(47.2%)	28(48.3%)	20(44.4%)	0.815
Female sex	79 (74.5%)	53 (91.4%)	23 (51.1%)	0.047
Height (m)	1.65 ± 0.09	1.62 ± 0.05	1.69 ± 0.04	0.106
Body surface area (m^2^)	1.56 ± 0.16	1.48 ± 0.09	1.66 ± 0.16	0.038
Projected EOAI (cm^2^/m^2^)	0.90 ± 0.04	0.95 ± 0.03	0.84 ± 0.02	0.041
Body mass index (kg/ m^2^)	22.9 ± 2.8	22.1 ± 1.1	22.9 ± 2.7	0.109
Pre-NYHA class				
I/II	52(49.1%)	35(60.3%)	16(35.6%)	0.097
III/IV	54 (50.9%)	23(39.7%)	29(64.4%)	0.105
Ejection fraction				
≥50%	45(44.3%)	30(51.7%)	15(33.3%)	0.159
<50%	58(55.7%)	28(48.3%)	30(66.7%)	0.207
Atrial fibrillation	15 (14.2%)	9 (15.5%)	4(8.9%)	0.281
Chronic heart failure	13(12.3%)	5(8.6%)	8(17.8%)	0.178
Ischemic heart disease	37(19.8%)	21(36.2%)	13(28.9%)	0.362
Renal insufficiency	8(7.5%)	5(8.6%)	3(6.7%)	0.518
Hypertension	36 (34.0%)	21(36.2%)	15(33.3%)	0.496
Diabetes	29 (27.3%)	10(17.2%)	16(35.6%)	0.079
Valve pathology				
Degenerative	33 (31.1%)	18 (31.0%)	14(31.1%)	0.557
Rheumatic	53 (50.0%)	27 (46.6%)	25(55.6%)	0.364
Infective	3 (2.8%)	2(3.4%)	1(2.2%)	0.599
CPB time (min)	108 ± 32	105 ± 21	110 ± 35	0.541
Cross-clamp time (min)	90 ± 23	87 ± 18	92 ± 27	0.617
In-hospital death	3(2.8%)	-	-	-

NYHA, New York Heart Association; CPB, cardiopulmonary bypass; EOAI, effective orifice area index; *Data were compared between the non-PPM group and the moderate PPM group.

### Echocardiographic measurements and calculations

Echocardiographic measurements included M-mode, two-dimensional, continuous wave, and Doppler analysis. Standard M-mode cardiac dimensions were collected according to the criteria of the American Society of Echocardiography. All Doppler measurements were obtained as the average of at least 3 cycles in patients with sinus rhythm or more than 5 cycles in those with atrial fibrillation. The following parameters were collected: end-diastolic septal thickness, left ventricular end-diastolic dimension, and end-diastolic left ventricular posterior wall thickness. The peak and mean transvalvular gradients were calculated by the complete Bernoulli equation. The mass of the left ventricle (LV) was estimated according to the joint recommendations of the American and European associations of echocardiography using Devereux’s [16] and indexed to BSA (LVMI). The effective orifice area (EOA) was determined by the standard continuity equation [(LVOT^2^ × 0.785 × TVI_1_)/TVI_2_)], where *LVOT* is the diameter of the left ventricular outflow tract, and *TVI*_*1*_ and *TVI*_*2*_ are the time-velocity integrals at the LVOT and across the aortic valve, respectively. The measured EOAi, a valid parameter for quantification of the severity of P-PtM, was calculated by dividing the EOA by the BSA. Also, the projected EOAi was derived from the published normal in vivo EOA values for the 17 mm SJMR valve and indexed to BSA [12]. Unless specified otherwise, EOAi throughout this article indicates measured EOAi.

### Operative techniques and anticoagulation therapy

All patients were approached through a median sternotomy. Cardiopulmonary bypass was initiated after cannulation of the ascending aorta, superior vena cava and inferior vena cava. The operation was performed with moderate hypothermia and use of antegrade cold blood cardioplegia. No patient underwent aortic annulus enlargement or root replacement. After removal of the diseased leaflets and calcifications from the annulus, the size of the prosthesis was determined according to the diameter of the aortic annulus, which was measured with manufacturer’s sizers (St. Jude Medical, USA). The 17-mm SJMR were then implanted in the supra-annular position using 2–0 interrupted polyester –non-everting mattress sutures in all patients. After the first postoperative day, patients received oral warfarin sodium at daily updated dosages according to the international normalized ratio of prothrombin time (PT-INR), with a target value maintained between 1.5 and 2.0.

### Follow-up

Clinical follow-up was updated to June 2013 through direct hospital visits and structured telephone interviews for all survivors. Both clinical and echocardiographic assessments were scheduled by protocol at discharge (mean 7.4 ± 2.1 days), sixth postoperative month and yearly thereafter. Follow-up transthoracic echocardiographic data were obtained for 96 (93.2%) of the 103 discharged patients at 45.8 ± 8.8 months after primary surgery. The clinical follow-up was 97.1% complete. Mean follow-up time was 52.6 ± 11.9 months (median, 50.5 months; range, 14-74 months). In case of new-onset symptoms, additional echo were performed, and the patient’s follow-up charts were updated accordingly. The valve-related complications were defined according to the guidelines for reporting after cardiac valve interventions [17].

### Statistical analysis

Continuous variables are presented as mean ± standard deviation and categorical data as percentages unless otherwise specified. Differences in pre- and postoperative echocardiographic data for all patients were compared and analyzed by paired Student’s t-test. Differences in patients with and without moderate P-PtM early after surgery were analyzed by χ^2^, Fisher exact or unpaired Student’s *t* test as appropriate. Pearson’s coefficient was used to analyze the correlation between the projected EOAi and the measured EOAi. The actuarial survival rate and ratio of patients without valve-related complications were calculated by the Kaplan-Meier method and compared between groups by using a log-rank test. Cox proportional-hazards regression model was used to determine whether the occurrence of moderate P-PtM was associated with increased risk of valve-related complications and intermediate-term mortality. Variables with a univariate *p* < 0.2 or those of known clinical importance for survival and adverse events were submitted to the multivariate models to calculate hazards ratios (HR) and its 95% confidence intervals (CI). All *p* values less than 0.05 were considered statistically significant. All statistical analyses were performed using Statistical Package for Social Sciences version 16.0 (SPSS Inc, Chicago, IL, USA).

## Results

### Operative morbidity and mortality

In-hospital mortality was 2.8% (3 patients). The causes of the in-hospital death were cerebral infarction (1 patient), respiratory failure (1 patient) and low cardiac output syndrome (1 patient). The early postoperative period was complicated in two patients by atrioventricular block requiring pacemaker implantation. Univariate analysis identified left ventricular ejection fraction (LVEF) <35% as an independent predictor of in-hospital mortality (HR 1.32, 95% CI: 1.09 to 1.78, p = 0.041). However, multivariable analysis yielded no significant predictor of early postoperative death. Two patients experienced perioperative intra-aortic balloon pumping for hemodynamic instability.

### Clinical follow-up and valve-related complications

**Figure 1:**
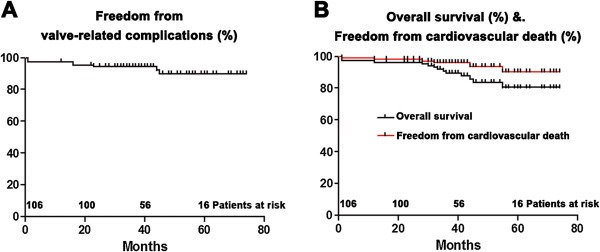
**(A) Freedom from valve**-**related complications; (B) Midterm survival and freedom from cardiac death.**

**Figure 2:**
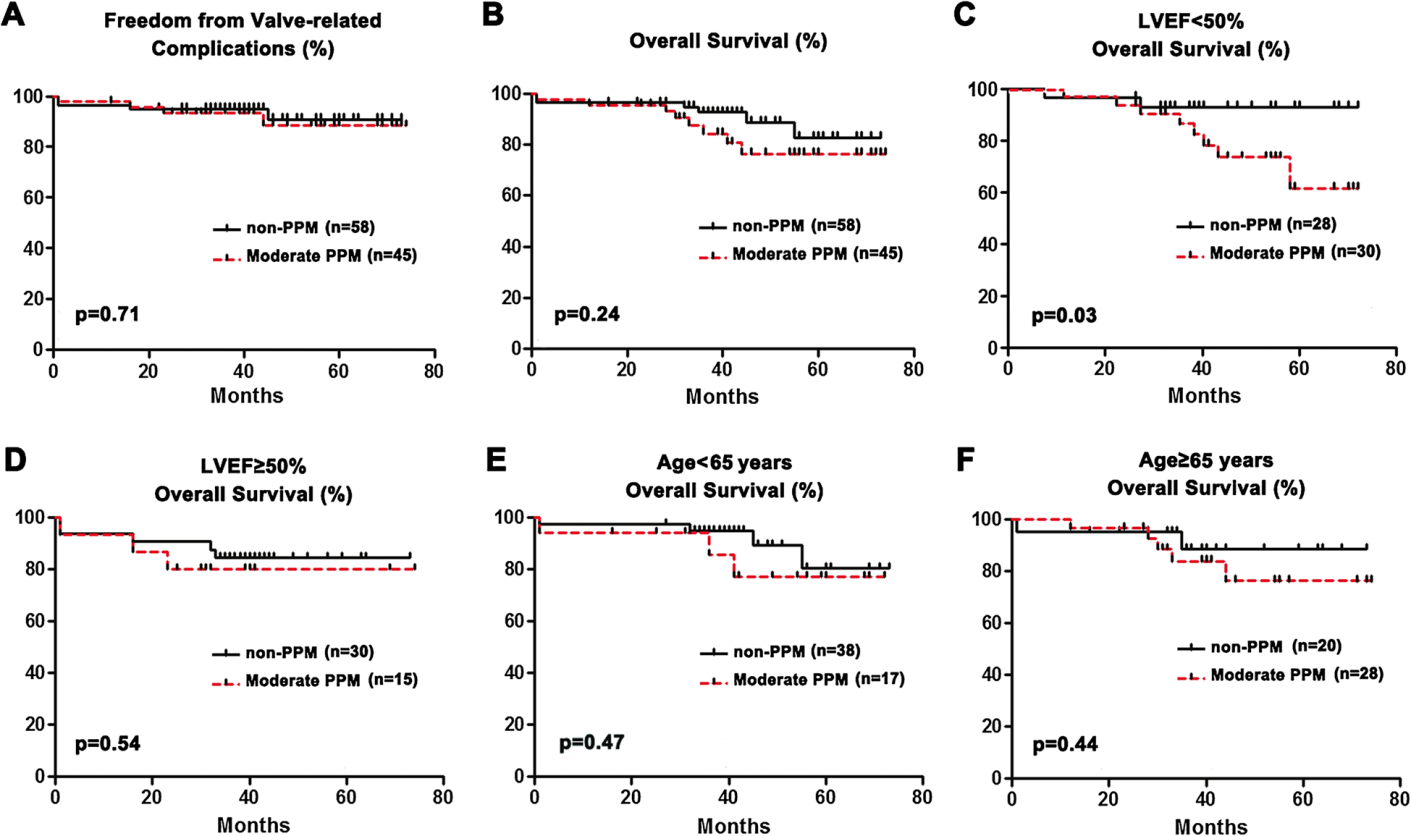
**Impact of moderate prosthesis**-**patient mismatch on postoperative outcomes: (A) Overall valve-related complications; (B) Overall survival; (C) Preoperative left ventricular ejection fraction (LVEF) <50%; (D) LVEF** ≥ **50%; (E) Patients < 65 years old; (F) Patients** ≥ **65 years old.**

During follow-up, NYHA functional class improved in the entire cohort and the improvement was not significantly different between the groups with and without moderate P-PtM. Three patients were readmitted due to prosthetic endocarditis, two patients were reoperated for paravalvular leakage, and one patient experienced cerebral hemorrhage. Late cerebral infarction, prosthetic thrombosis, and structural failure of the mechanical valves did not occur in any patient during follow-up. Freedom from valve-related complications at 1-year, 3-year, and 5-year was 97.2%, 94.3%, and 89.9%, respectively (Figure 1A). Univariate analysis identified atrial fibrillation, diabetes and renal insufficiency were associated with an increased risk of postoperative valve-related complications, and only diabetes was identified as the independent predictor of valve-related complications by multivariate analysis (Table 2). Freedom from reoperation at an average of 52.6 ± 11.9 months after primary AVR was 98.0%. Long-term survival and freedom from cardiovascular death are shown in Figure 1B. Of the 103 survivors, 12 patients (10.6%) died during the follow-up period: myocardium infarction (n = 2), malignancy (n = 2), heart failure (n = 2), sepsis (n = 2), pneumonia (n = 1), renal failure (n = 1), unknown (n = 2). Actuarial 1-year, 3-year, and 5-year survival rate were 96.2%, 89.4%, and 80.5%, respectively. Univariate analysis revealed age, preoperative LVEF <35%, diabetes, renal insufficiency, and a concomitant coronary surgery as predictors of overall mortality (including in-hospital death). Multivariate analysis showed diabetes and preoperative LVEF <35% to be the independent predictors of the postoperative death (Figure 2). Freedom from cardiovascular death at 1-year, 3-year, and 5-year was 98.1%, 95.9%, and 90.2%, respectively. As for the 8 patients with severe P-PtM at discharge, only one anticoagulation-related complication was observed and no cardiovascular death was observed during an average of 48.7 ±8.9 months follow-up.

**Table 2:** **Univariate and multivariate analysis for independent predictors of valve**-**related complications and mortality**

**Variables**	**Valve-related complications**		**Overall mortality**	
	**Univariate**	**Multivariate**	**Univariate**	**Multivariate**
	**(P value)**	**HR[95% CI], P value**	**(P value)**	**HR[95% CI], P value**
< 65 years	0.165	3.4[0.6-8.1], 0.320	0.108	1.6[0.8-2.7], 0.245
≥ 65years	0.087	0.9[0.2-1.6], 0.194	0.047	1.3[1.0-1.7], 0.071
Sex: male	0.548	-	0.876	-
Body mass index	0.265	-	0.178	-
I/II	0.374	-	0.217	-
III/IV	0.081	1.5[0.9-2.8], 0.075	0.098	1.4[1.0-1.6], 0.059
>50%	0.727	-	0.324	-
35%-50%	0.165	2.0[1.1-3.7], 0.234	0.061	1.3[0.9-1.7], 0.088
<35%	0.071	1.3[0.7-1.9], 0.128	<0.001	2.0[1.1-3.7], 0.021
>0.85 cm^2^/m^2^	0.791	-	0.564	-
>0.65 to <0.85 cm^2^/m^2^	0.108	1.2[0.9-1.4], 0.144	0.145	1.2[0.9-1.6], 0.188
Atrial fibrillation	0.013	1.1[0.5-2.7], 0.088	0.076	1.3[0.2-2.2], 0.064
Congestive heart failure	0.102	1.6[0.7-2.9], 0.268	0.128	2.2[0.6-2.1], 0.091
Renal insufficiency	0.041	2.3[0.8-4.3], 0.099	0.027	1.6[0.3-3.3], 0.054
Hypertension	0.323	-	0.279	-
Diabetes mellitus	<0.001	3.2[1.8-6.4], 0.015	<0.001	3.8[2.0-5.9], 0.002
Chronic lung disease	0.094	1.7[0.6-3.3], 0.102	0.211	1.6[0.1-1.8], 0.069
Concomitant CABG	0.072	1.6[0.8-4.1], 0.109	0.033	3.1[0.9-8.7], 0.084
CPB time (min)	0.382	1.0[0.8-1.2], 0.502	0.089	1.6[0.8-2.3], 0.069
Cross-clamp time (min)	0.687	1.0[0.3-3.5], 0.751	0.139	1.5[1.1-2.1], 0.078

CABG, coronary artery bypass grafting; CI, confidence intervals; HR, hazard ratio; other abbreviations as in Tables 1.

### Echocardiographic follow-up

The baseline and postoperative echocardiographic data of all patients are reported in Table 3. A significant reduction in peak and mean transvalvular gradient was observed in all patients, with a mean difference versus preoperative values of 82.9 ± 29.5 mmHg and 50.2 ± 16.8 mmHg, respectively. Follow-up echocardiography also revealed a significant regression of LVMI, decreasing from preoperative values of 196.1 ± 44.3 g/m^2^ to 118.9 ± 30.8 g/m^2^ after surgery. During follow-up, the mean EOA and EOAi were significantly increased compared with preoperative values. No significant difference between preoperative and postoperative LVEF was observed.

**Table 3:** **Echocardiographic preoperative and post**-**operative data**

**Variables**	**Overall**	**Non-PPM**	**Moderate PPM**	***p *** value*
	**(n = 103)**	**(n = 58)**	**(n = 45)**	
**Preoperative data**				
Peak TVG (mmHg)	115.2 ± 38.7	121.4 ± 36.9	107.2 ± 31.8	0.362
Mean TVG (mmHg)	68.1 ± 21.9	71.2 ± 18.79	64.1 ± 26.8	0.381
LVMI (g/m^2^)	196.1 ± 44.3	201.7 ± 54.6	188.8 ± 68.4	0.154
EOAI (cm^2^/m^2^)	0.44 ± 0.18	0.42 ± 0.16	0.47 ± 0.08	0.631
Ejection fraction (%)	58.4 ± 11.9	59.8 ± 12.6	56.6 ± 9.9	0.184
**Postoperative (at discharge) data**				
Peak TVG (mmHg)	^a^31.8 ± 12.1	^a^28.7 ± 13.4	^a^35.8 ± 16.1	0.398
Mean TVG (mmHg)	^a^17.2 ± 5.98	^a^16.3 ± 4.69	^a^18.4 ± 7.9	0.691
LVMI (g/m^2^)	170.8 ± 48.9	167.1 ± 39.1	175.6 ± 54.3	0.437
EOAI (cm^2^/m^2^)	^a^0.91 ± 0.21	^a^1.01 ± 0.13	^a^0.78 ± 0.09	<**0.01**
Ejection fraction (%)	56.9 ± 15.4	58.1 ± 13.4	55.3 ± 10.9	0.697
**Follow-up data**				
Peak TVG (mmHg)	^b^32.3 ± 8.91	^b^29.6 ± 7.4	^b^35.1 ± 11.4	0.412
Mean TVG (mmHg)	^b^17.9 ± 6.79	^b^16.8 ± 5.14	^b^19.3 ± 8.16	0.689
LVMI (g/m^2^)	^b^118.9 ± 30.8	^b^117.1 ± 26.1	^b^121.2 ± 33.8	0.186
EOAI (cm^2^/m^2^)	^b^0.96 ± 0.18	^b^0.99 ± 0.21	^b^0.92 ± 0.16	0.781
Ejection fraction (%)	60.1 ± 6.5	61.2 ± 7.1	58.7 ± 10.3	0.336
**Reduction rate (%): preoperative to follow-up**				
Peak TVG (%)	72.0 ± 12.6	75.6 ± 9.9	67.3 ± 15.9	0.131
Mean TVG (%)	73.7 ± 8.9	76.4 ± 13.1	69.8 ± 12.5	0.152
LVMI (%)	39.4 ± 20.9	41.9 ± 18.4	35.8 ± 19.8	0.109

TVG, transvalvular gradients; LVMI, left ventricular mass index; other abbreviations as in Tables 1 and 2. *Data were compared between the non-PPM versus the moderate PPM; ^a^*P* < 0.05 for postoperative (at discharge) data versus preoperative data; ^b^*P* < 0.05 for follow-up data versus preoperative data.

### Relationship between the projected EOAi and measured EOAi

The projected EOAi of the two groups was calculated and significant difference between groups was observed (Table 1). A significant correlation between the projected EOAi and measured EOAi was observed (r = 0.74, p = 0.03). However, in the subgroup analysis of patients with LVEF < 50% (n = 58), the projected EOAi moderately correlated with the measured EOAi (r = 0.59, p = 0.051), and the projected EOAi has a sensitivity of 74% and specificity of 55% for predicting moderate P-PtM postoperatively.

### Effects of P-PtM on clinical and echocardiographic variables

The prevalence of moderate P-PtM, defined as an EOAI ranging from 0.65 cm^2^/m^2^ to 0.85 cm^2^/m^2^, was documented in 46 patients at discharge (43.4%). In comparison to the patients without P-PtM, the group of patients with moderate P-PtM had a significantly greater mean BSA, proportion of males and bicuspid aortic valve disease (Table 1). As demonstrated in Table 3, the EOAI at discharge were significantly lower in patients with moderate P-PtM than in those without P-PtM. During the follow-up period, no difference in the regression of LVMI, decrease of transvalvular pressure gradients, valve-related complications and overall survival (Figure 2A and Figure 2B) between groups was observed. However, moderate P-PtM was associated with increased midterm mortality in patients with LV ejection fraction < 50% (HR 1.80, 95% CI: 1.32 to 2.46, p = 0.02), but not in patients with preserved LV systolic function (Figure 2C and Figure 2D). Young age (< 65 years) was not associated with an increased risk of midterm mortality in patients with moderate P-PtM at discharge (Figure 2E and Figure 2F).

## Discussion

The present study demonstrated satisfactory outcomes of AVR with 17-mm SJMR valve in terms of midterm survival, physical capacity and hemodynamic performances. The data of our study corroborate with previous studies indicating the presence of moderate P-PtM early after surgery was not a risk factor of intermediate mortality. However, in our subgroup analysis, moderate P-PtM was associated with a significant decrease in overall survival in patients with impaired LV systolic function. These findings emphasize the importance of a prospective strategy for selection of appropriate AVR candidate and operative methods to achieve satisfactory results in patients with small aortic annulus.

Although the 17-mm SJMR valve is a new generation of aortic prosthesis with an improved hemodynamic profile, the beneficial effects of implanting this mechanical valve in patients with small aortic roots remain unclear, particularly in young patients and in the mid- to long-term. Okamura group reported a cohort of elderly patients (mean age >70 years) demonstrating satisfactory clinical improvement and regression of LVM after AVR with the 17-mm SJMR [2,10,11]. Similarly, a series of studies demonstrated favorable clinical outcomes, as well as improved hemodynamic performance both at rest and under an exercise load [2,8-11,18,19], after AVR with a 17-mm SJMR valve. However, these studies are mostly presented with a small number of elderly patients during a relatively short follow-up period. As the moderate P-PtM frequently occurred after implantation of a small aortic Regent valve [1,2,9-11], its potential adverse effects on postoperative outcomes are not well characterized. Therefore, we need to investigate furtherly into the safety and effectiveness of the 17-mm SJMR in a large number of patients of all ages.

Apart from its improved hemodynamic performances, the 17-mm SJMR valve was applied in this population for several other reasons. First, our patients presented with a relatively high-risk characteristics [high proportion of female patients (74.5%), NYHA class III/IV (50.9%), LVEF < 50% (55.7%), and concomitant coronary surgery (34.9%)], and therefore a “quick and simple” procedure was required to reduce myocardial ischemic time. It is clinically evident that a prolonged aortic cross-clamp time is one of the most important risk factors for post-AVR adverse events [3,4,6,7]. Although aortic root enlargement procedures or stentless prosthesis implantation are feasible options for patients with small aortic annulus, these procedures are technically more difficult and time consuming, and may result in higher morbidity than simple valve replacement, particularly in our patients who often have a severe calcified aortic root due to rheumatic and degenerative etiologies (81.1%). Moreover, a series of studies have clearly indicated that a better hemodynamic outcome early after surgery in patients underwent aortic root enlargement and AVR could not translate into an improved postoperative survival compared with patients with isolated AVR [2-4].

An important finding of this study is that moderate P-PtM is associated with increased postoperative mortality in patients with LVEF < 50%, but with normal prognosis in those with preserved ventricular function. Since patients with impaired LV function are more vulnerable to the excessive afterload imposed by P-PtM, it is reasonable to find in our series that the follow-up mortality are significantly increased in patients with a combination of moderate P-PtM and impaired LV systolic function. Studies from several groups also clearly indicated that the influential role of moderate P-PtM on late survival is more important in patients with reduced ventricular reserve than in those with preserved LV function [12,13]. On the contrary, however, some others failed to identify the moderate P-PtM as an independent predictor of valve-related complication and mortality after AVR [15,19]. These compelling evidence may be partially explained by the fact that investigators used different parameters (eg. projected, geometric or in vivo measured EOAi) to identify P-PtM and quantify its severity. As demonstrated in the present study, using the projected EOAi in predicting P-PtM in patients with LVEF < 50% has a lower accuracy; therefore, the projected EOAi to determine prediction and outcomes of P-PtM is of limited value. Also, differences in baseline characteristics and prevalence of moderate versus severe P-PtM in the patient population, as well as the diversity of the implanted prosthesis and surgical approaches are believed to contribute.

Previous studies have demonstrated that the influence of P-PtM on postoperative outcomes is an age-dependent phenomenon. As reported by Mohty et al. [12] and Moon et al. [20], moderate -to- severe P-PTM was detrimental to survival in young patients (<60 or 70 years of age), but its impacts on older patients are negligible. These findings might be related to the fact that younger patients are generally more physically active and they indeed have greater cardiac output requirements in relation to body size. However, in our series, younger age (<65 years) was not identified as an independent risk factor of increased midterm mortality and valve-related complications in patients with moderate P-PtM. A possible explanation could be that the proportion of patients with bioprosthetic valve in previous studies was as high as 67%-77.8% [12,13]. As P-PtM is a significant risk factor for accelerated degeneration of bioprosthetic heart valves [21], and bioprosthesis in younger patients may expose to the progression of valvular degeneration for a longer period of time, it seems very likely that the impact of moderate P-PtM on postoperative outcomes is more pronounced in younger patients than in older ones. In addition, our insignificant result might be owing to the limited numbers of the subgroup populations.

Some could argue that the use of mechanical prostheses in AVR may potentially increase mortality and morbidity compared with the use of bioprosthesis, particularly due to anticoagulation–related complications [22]. However, in the present study, which employed strict control of PT-INR within a range of 1.5-2.0, only three patients experienced anticoagulation-related adverse events, accounting for a linearized rate of 0.2% per patient-year. These complication rates are comparable to other reports of patients who underwent bioprostheses implantation [2,12,13,15]. According to the American College of Chest Physicians guidelines [23], our anticoagulant level is lower but is proved efficient in Chinese patients with mechanical heart valve prostheses [24,25]. Similarly, investigators from other Asian countries have demonstrated low incidence of thrombosis and hemorrhage complications with low intensity anticoagulant strategy, suggesting that mechanical valves are not a risk factor for postoperative adverse events with good control of PT-INR levels (1.8-2.2) [2,9].

### Study limitations

This study was subject to the limitations inherent to a retrospective, nonrandomized comparison of clinical data, so it is likely that a selection bias or unidentified confounders might have influenced the results. In the present study, moderate P-PtM was determined by echocardiographic-measured EOAi. As suggested by other studies [8,26], the utilization of the measured EOAi to define P-PtM may lead to an underestimation of the impact of P-PtM on survival. Hence, it is reasonable that no significant impact of P-PtM on postoperative mortality was observed in our series. Moreover, the measurement of LVMI in our study was performed by M-mode echocardiography, which is regarded less accurate than magnetic resonance imaging.

The improvement of a patient’s physical capacity was not quantified in the present study (eg. the use of the SF-36 questionnaire), and the impact of the moderate P-PtM on patients’ quality of life is thereby questionable. Beyond the preoperative LVEF and age, body mass index is also an important factor that may have a significant impact on postoperative outcomes. However, in our series, only a small proportion of patients (11 patients) have a body mass index above 25 kg/m^2^. Hence, the observed effects of the moderate P-PtM on midterm outcomes after AVR with a 17-mm Regent valve cannot be generalized to obese patients. In addition, the small number of patients with severe P-PtM in our series prevented our further investigation of the long-term safety and effectiveness of the 17-mm Regent valve.

## Conclusions

Moderate P-PtM after implantation of a small mechanical prosthesis at aortic position is associated with increased midterm mortality and valve-related complications in patients with pre-existing LV dysfunction. Selected patients with small aortic annulus can experience satisfactory clinical results and midterm survival after isolated AVR with a 17-mm SJMR valve.

## Abbreviations

AVR: Aortic valve replacement; BSA: Body surface area; EOA(i): Effective orifice area (index); LV: Left ventricle; LVM(i): Left ventricular mass (index); LVEF: Left ventricular ejection fraction; NYHA: New York Heart Association; P-PtM: Prosthesis-patient mismatch; PT-INR: International normalized ratio of prothrombin time; SJMR: St. Jude Medical Regent.

## Competing interests

The authors declare that they have no competing interests.

## Authors’ contributions

JH designed the protocol, analyzed data, contributed to the discussion and wrote the manuscript. HQ, YL and JG collected, researched data, contributed to the discussion. JJZ analyzed and researched data. EZ contributed to the discussion and reviewed/edited the manuscript. All authors read and approved the final manuscript.
